# Bilingual Language Switching: Production vs. Recognition

**DOI:** 10.3389/fpsyg.2017.00934

**Published:** 2017-06-07

**Authors:** Michela Mosca, Kees de Bot

**Affiliations:** ^1^International Doctorate for Experimental Approaches to Language and Brain (IDEALAB), University of Potsdam, Potsdam, Germany / University of Groningen, Groningen, Netherlands / University of Trento, Trento, Italy / University of Newcastle, Newcastle upon Tyne, United Kingdom / Macquarie UniversitySydney, NSW, Australia; ^2^Potsdam Research Institute for Multilingualism, University of PotsdamPotsdam, Germany; ^3^Department of Applied Linguistics, University of PannoniaVeszprém, Hungary

**Keywords:** language switching, IC model, BIA model, bilingual production and recognition, language inhibition

## Abstract

This study aims at assessing how bilinguals select words in the appropriate language in production and recognition while minimizing interference from the non-appropriate language. Two prominent models are considered which assume that when one language is in use, the other is suppressed. The Inhibitory Control (IC) model suggests that, in both production and recognition, the amount of inhibition on the non-target language is greater for the stronger compared to the weaker language. In contrast, the Bilingual Interactive Activation (BIA) model proposes that, in language recognition, the amount of inhibition on the weaker language is stronger than otherwise. To investigate whether bilingual language production and recognition can be accounted for by a single model of bilingual processing, we tested a group of native speakers of Dutch (L1), advanced speakers of English (L2) in a bilingual recognition and production task. Specifically, language switching costs were measured while participants performed a lexical decision (recognition) and a picture naming (production) task involving language switching. Results suggest that while in language recognition the amount of inhibition applied to the non-appropriate language increases along with its dominance as predicted by the IC model, in production the amount of inhibition applied to the non-relevant language is not related to language dominance, but rather it may be modulated by speakers' unconscious strategies to foster the weaker language. This difference indicates that bilingual language recognition and production might rely on different processing mechanisms and cannot be accounted within one of the existing models of bilingual language processing.

## Introduction

When a speaker of more than one language (hereafter “bilingual”) processes a language, words from the non-relevant language might be activated and interfere. This can happen while speaking, but also during writing, listening and reading. The ability to confine processing to the relevant language is called “language control” and is essential for successful communication. Despite the importance of this phenomenon, research on language control has predominantly concentrated on language production (e.g., Meuter and Allport, 1999; Jackson et al., 2001; Costa and Santesteban, 2004; La Heij, 2005; Finkbeiner et al., 2006; Abutalebi and Green, 2007, 2008; Gollan and Ferreira, 2009; Calabria et al., 2012; Linck et al., 2012; Filippi et al., 2014; Goldrick et al., 2014), while much less attention has been devoted to language recognition (e.g., Grainger and Beauvillain, 1987; von Studnitz and Green, 1997, 2002; Thomas and Allport, 2000; Orfanidou and Sumner, 2005; Wang, 2015). Moreover, language production and recognition have been often investigated separately, leaving unclear whether the two processes rely on the same or different mechanisms. The present paper focusses on bilingual language control in production and recognition.

### Language control in production and recognition

To investigate language control, most of the studies have focused their attention on spoken (but not written) word production and visual (but not spoken) word recognition. In spoken word production, language control refers to the capacity of a bilingual person to speak in the intended language, while avoiding interferences from the non-intended language. In visual word recognition, language control indicates the ability to understand the meaning of written words belonging to a certain language, while reducing interference from the non-target language. These processes are far from being effortless. Indeed, it is generally agreed that when a word from the intended language is processed, also words from the irrelevant language are coactivated and might interfere during processing (e.g., for production: Poulisse and Bongaerts, 1994; Hermans et al., 1998; Colomé, 2001; Kroll et al., 2006; for recognition: Dijkstra et al., 1998, 2000; van Heuven et al., 1998). Much evidence has suggested that interference from the irrelevant language might be resolved by suppressing words of the non-target language (e.g., for production (Green, 1998); for recognition: (Dijkstra and van Heuven, 1998, 2002; van Heuven et al., 1998); but see La Heij, 2005; Costa et al., 2006; Finkbeiner et al., 2006; Kroll et al., 2008). According to the Inhibitory Control (IC) model proposed by Green (1986, 1993, 1998), the amount of inhibition applied to the non-relevant language depends on its dominance. This means that a greater magnitude of inhibition is needed to suppress the stronger language (e.g., the native language or “L1”) compared to the weaker language (e.g., a later acquired language or “L2”). It follows that the cost to reactivate a language depends on the strength of inhibition previously applied, with more strongly inhibited languages having larger reactivation costs than less strongly inhibited languages. Hence, within the IC model reactivation costs (also “switching costs”) are predicted to be larger for the stronger than for the weaker language. In this framework, switching costs are *dominance-related*.

Even though the predictions of the IC model have been mainly used to investigate language control in production, the model was conceived to be applicable to both bilingual production and recognition (e.g., Green, 1986, 1998). Research on bilingual recognition, however, has mainly relied on a computational model called Bilingual Interactive Activation (BIA) (Grainger and Dijkstra, 1992; Dijkstra and van Heuven, 1998; van Heuven et al., 1998). According to the BIA, when a word is presented, similar words from both the relevant and the irrelevant language are activated. The activated words send activation to the respective language node (representational layer containing language tags). Language competition is solved via inhibition from the language node to the words of the other language. The amount of inhibition applied to the words of the other language depends on the strength of activation of the language node. Specifically, the stronger the activation of the language node the greater the inhibition of the words of the other language, i.e., “asymmetric inhibition.” Because of this, words from the non-relevant language are more strongly inhibited than words from the relevant language. In this way, the word of the relevant language that best matches the input becomes most active and crosses the “recognition threshold” (Dijkstra and van Heuven, 1998). More generally, the BIA suggests that the activation of the language node reflects the amount of activity in the lexicon. Since L1 words have a higher baseline activation level than L2 words, the L1 language node is more activated than the L2 language node, and inhibition is greater on L2 than on L1 words (Dijkstra and van Heuven, 1998; van Heuven et al., 1998). Therefore, if inhibition is asymmetrical, namely greater for L2 words than for L1 words, then also switching costs for the L2 and the L1 are expected to be asymmetrical, that is larger for the weaker L2 than for the stronger L1. In this scenario, switching costs are *dominance-reversed*^1^. Similar conclusions concerning language recognition within the BIA model were provided by Grainger et al. (2010), suggesting that since L1 words have a higher resting level of activation than L2 words, on a switch trial interference from the L1 into the L2 is greater than otherwise, leading to larger costs for the weaker than for the stronger language.

Briefly, the different predictions with regard to the IC and the BIA model described in this paper derive from the fact that the amount of inhibition depends on the activation level of the words of the *non-relevant language* (words of the stronger language are inhibited more than words of the weaker language) in the former and on the activation level of the language node of the *relevant language* (language node of the stronger language inhibits words of the weaker language to a greater extent than vice versa) in the latter.

### Language switching

To measure switching costs and thus cast light on how bilinguals control their languages, a language switching paradigm is required. The language switching paradigm includes two types of trials, *repetition trials* (stimuli in the same language as in the preceding trial, e.g., L1-L1) and *switch trial*s (stimuli in a different language compared to the preceding trial, e.g., L2-L1). Responses on switch trials are usually less accurate and slower compared to repetition trials, and this difference is known as language “switching costs” (e.g., Meuter and Allport, 1999).

Several studies on bilingual language control in production have interpreted larger switching costs for the stronger than for the weaker language, i.e., *asymmetrical switching costs*, as evidence for dominance-related inhibition as predicted by the IC model (e.g., Meuter and Allport, 1999; Jackson et al., 2001; Macizo et al., 2012; but see Bobb and Wodniecka, 2013; Peeters et al., 2014; Fink and Goldrick, 2015; Reynolds et al., 2016). However, it has been also shown that when the dominance difference between the L1 and the L2 is relatively small, the amount of switching costs for the two languages becomes comparable, i.e., *symmetrical switching costs* (e.g., Christoffels et al., 2007; Schwieter and Sunderman, 2008; Declerck et al., 2013; Fink and Goldrick, 2015). Based on this, the predictions of the IC model have been expanded by suggesting that when the dominance difference between two languages is relatively small, the amount of inhibition applied to the two languages is similar, yielding comparable switching costs (e.g., Meuter and Allport, 1999; but see Costa and Santesteban, 2004; Costa et al., 2006; Verhoef et al., 2009; for alternative explanations).

As far as recognition is concerned, few studies have investigated how bilinguals control languages (e.g., von Studnitz and Green, 1997; Thomas and Allport, 2000; for a review on bilingual word recognition see van Assche et al., 2012). Interestingly enough, most of the studies reported a similar magnitude of switching costs for the stronger L1 and the weaker L2, that is symmetrical switching costs (e.g., for lexical decision task: von Studnitz and Green, 1997; Thomas and Allport, 2000; Orfanidou and Sumner, 2005; for categorization task: von Studnitz and Green, 2002; Macizo et al., 2012; but see Jackson et al., 2004). Unfortunately, symmetrical switching costs do not clearly indicate whether bilingual language control in recognition can be better accounted for by the IC or the BIA model. Indeed, it is the direction of switching costs asymmetry (whether switching costs are larger for the stronger or for the weaker language) that more clearly indicates whether language control in recognition can be better explained by the IC model (predicting larger switching costs for the stronger than for the weaker language) or the BIA model (predicting larger costs for the weaker than for the stronger language). Furthermore, these results have led to the question whether the preponderant presence of asymmetrical switching costs in production relative to recognition tasks is due to methodological inconsistencies across studies or to the fact that bilingual language control in production and recognition rely on two different mechanisms (Reynolds et al., 2016). According to Reynolds et al. (2016), the difference usually found between the two modalities could be attributed to the fact that language production and recognition differ in terms of the specific systems required to perform the task. For example, in picture naming, after the semantic information of the picture has been retrieved, its phonological representation has to be activated, as to allow its articulation. In this sense, language production strongly relies on phonological encoding. However, during visual word recognition, most of the attention is devoted to decoding the orthographic form of the word. After the corresponding word has been found in the lexicon, activation is sent to its semantic information. In this sense, visual word recognition predominantly relies on orthographic decoding. Since phonological encoding is supposed to be a more demanding process compared to orthographic decoding (Reynolds and Besner, 2006), the former is expected to be more susceptible to interference from the other language than the latter. In this scenario, language production is more sensitive to languages' activation level than language recognition is. Even though the details of the two processes are not specified, the authors suggest that this would lead to larger switching costs for the stronger than for the weaker language in language production and to a comparable amount of switching costs in language recognition. Therefore, according to the authors, language control in production and recognition is supported by different mechanisms. A similar view is held by Macizo et al. (2012), suggesting that different mechanisms underpin bilingual language production and recognition. In particular, they argued that bilingual language production is achieved by reactively inhibiting the competing language (the stronger the competing language, the greater the inhibition applied to it), whilst in bilingual recognition, such a competition is not present and inhibition of the non-relevant language is not required. However, while this view can account for the asymmetrical switching costs found in the production studies involving language switching, it leaves unclear what the source of the language switching costs in bilingual recognition studies is. Evidence in favor of the hypothesis that bilingual language production and recognition may rely on different processes comes from a study using magnetoencephalography (MEG) to investigate the degree of overlap between language control in production and recognition (Blanco-Elorrieta and Pylkkänen, 2016). This study revealed an anatomical dissociation between the areas supporting language switching in production and recognition. Whilst in production the switch effect was localized in the dorsolateral areas of the prefrontal cortex (dlPFC), in recognition the same effect was ascribed to the anterior cingulate cortex (ACC). These results led the authors to conclude that the strategies used during language control might be influenced by whether the language switch is active (e.g., in production) or passive (e.g., in recognition). In contrast to this, other studies have proposed that overlapping processes might support language control in production and recognition. For example, Peeters et al. (2014) found “comprehension to production language switching costs,” that is naming pictures in the L1 (production task) was slower when the preceding word belonged to the L2 rather than to the L1 (comprehension task). The result that language switching costs can arise across modalities led the authors to propose that language production and recognition are supported by a unique mechanism of language control. Gambi and Hartsuiker (2016) went a step further by observing that language switching costs across modalities can arise also when the two tasks are performed by different speakers. Specifically, the authors found that passive listening to L2 words produced by another speaker caused language switching costs during the production of L1 words. Based on this outcome, the authors concluded that language control in production and recognition is supported by shared mechanisms.

The goal of the present study is to shed light on this controversy. More precisely, the study aims at investigating whether (1) language control in recognition is dominance-related (as the IC model predicts) or dominance-reversed (as the BIA model predicts) and from this at assessing, more generally, whether (2) language control in bilingual recognition and production rely on the same mechanisms. To do it, we tested one group of unbalanced bilinguals (advanced L2 speakers, with a stronger L1 and a weaker L2) in both a recognition and a production task involving language switching. As to recognition, participants were administered a bilingual lexical decision task; regarding production, participants performed a bilingual picture naming task. In both tasks, switching costs in L1 and L2 were measured. Moreover, to assess languages' baseline strength of activation (believed to be a reflection of language dominance), a “single-language” block was included in the picture naming task, in which pictures had to be named in either L1 or L2 separately.

Concerning the predictions, we expect one of two possible outcomes: (1) If the amount of inhibition of the non-relevant language strongly depends on language dominance, we expect to find asymmetrical switching costs in both the lexical decision and the picture naming task. In this framework, if the magnitude of inhibition is dominance-related (as the IC model predicts), then switching costs are expected to be larger for switching to the stronger than for switching to the weaker language in both tasks. If, however, language inhibition in recognition is dominance-reversed (as predicted by the BIA model), switching costs in the lexical decision tasks are expected to be larger for the weaker than for the stronger language. (2) Alternatively, if asymmetric inhibition is applied only in cases of extreme dominance difference between two languages (such in the case of low proficient L2 speakers), then switching costs are expected to be symmetrical in both the recognition and the production task.

## Participants

For the present study, we recruited 32 native speakers of Dutch (6 men, mean age: 21.8 years, sd: 4.49) from the student population of the University of Groningen (mean years of formal education: 16.7, sd: 1.93). Participants were tested in Dutch and English and were paid for their participation. All participants were right-handed, had normal or corrected to normal vision and had never been diagnosed with reading, learning or language disability. Before the experiment, participants gave their written consent and filled out the Language Experience and Proficiency Questionnaire (LEAP-Q, Marian et al., 2007) to assess their language profiles.

All participants had acquired Dutch from birth as the native language (L1) and English at school as a second language (L2) for a minimum of 6 years (mean L2 AoA: 9.35 years, sd: 2.34). On a daily basis, speakers were mostly exposed more to Dutch (57%) than to English (34%) or to other languages (9%). To screen for language skills, participants were asked to self-rate their ability to speak, understand and read in Dutch and English based on a ten-point scale, where 0 = no knowledge and 10 = perfect knowledge. Overall, results revealed that participants considered themselves excellent users of Dutch (mean score: 9.16, sd: 0.85) and good users of English (mean score: 7.65, sd: 1.04). Their L2 proficiency level was tested using the grammar part of the paper-based Oxford Placement Test (Allan, 2004). The test yielded a mean score of 84.4% (sd: 4.72) correct answers, indicating that participants were highly proficient L2 users (C1-C2 level), according to the Common European Framework of Reference for Languages (CEFR, Council of Europe, 2001). For detailed information on participants, see Appendix A in Supplementary Data.

## Materials

Stimuli of the lexical decision task were 28 words representing simple concrete objects and 28 non-existing words, i.e., pseudowords. With regard to the words, half of the items were in Dutch and the other half were their English translations^2^. Based on the information of the webCELEX database (http://celex.mpi.nl/), words were matched for word form and lemma frequency (*t* = 1.49, *p* > 0.05 and *t* = 1.25, *p* > 0.05) as well as for letter orthographic length (*t* = 1.22, *p* > 0.05). Words were also matched according to their orthographic neighborhood density within each language (*t* = 1.54, *p* > 0.05), between the two languages (*t* = 0.75, *p* > 0.05) and across other languages (German, *t* = 0.47, *p* > 0.05; French, *t* = 1.18, *p* > 0.05 and Spanish, *t* = 1.32, *p* > 0.05). The values for the orthographic neighborhood density were taken from the Cross Linguistic Easy Access Resource for Phonological and Orthographic Neighborhood Densities database (CLEARPOND; Marian et al., 2012). Furthermore, we made sure that words were legal string of letters in both Dutch and English. To do it, we checked that all bigram transitions and single letter positions were probable and that probability was comparable in the two languages. Finally, we did not include cognates, homophones or words belonging to the same semantic category.

With concern to pseudowords, they were created by modifying the first or the last subsyllabic elements of the words selected for the lexical decision task. Apart from this change, pseudowords maintained the subsyllabic structure of the words they were generated from (e.g., from the words “glass-es” and “wo-lk” the pseudowords “smart-es” and “wo-lm” were derived). Half of the pseudowords were created starting from the selected Dutch words and the other half from the selected English words by using the multilingual pseudoword generator Wuggy (Keuleers and Brysbaert, 2010). To refrain participants from relying on subtle cues to differentiate between words and pseudowords, we matched words and pseudowords on several levels. Specifically, all pseudowords obeyed to the phonotactic constraints of both Dutch and English (i.e., they were legal string of letters in both languages) and they had an overlap ratio of 2/3 to existing words (i.e., they looked and sounded like real words). To make sure that the generated pseudowords equally represented words in Dutch and English, they were matched as closely as possible according to their bigram transitions and single letter positional probability in the two languages. Moreover, pseudowords were matched according to their orthographic Levenshtein distance to real words in the lexicon (*t* = 0.31, *p* > 0.05). The neighborhood size difference between pseudowords and words was kept as minimal as possible (neighborhood size difference <0.45, see also Keuleers and Brysbaert, 2010) and this was matched across pseudowords (*t* = 0.51, *p* > 0.05). Finally, words and pseudowords had the same orthographic length.

For the picture naming task, we used 14 pictures corresponding to the words used in the lexical decision task. Pictures were taken from the “Colorized Snodgrass and Vanderwart pictures” set (Rossion and Pourtois, 2004) and had to be named either in Dutch or in English (leading to a total of 28 words, the same used in the lexical decision task).

All items were presented in the center of a 15-inch computer screen set to 1,280 × 800 pixel resolution and they were seen from a distance of approximately 80 cm. Words and pseudowords were presented in white lowercase letters (font: Courier New, point size: 36) against a black background. Pictures had a size of 197 × 281 pixel and were presented against a colored (green or blue) background. Stimuli were presented using the software E-Prime Professional version 2.0 (Psychology Software Tools, Pittsburgh, PA; Psychology Software Tools Inc., 2012). For detailed information on the stimuli used See Appendix B in Supplementary Data.

## Procedure

Participants were tested individually in a quiet laboratory room. They were first given verbal instructions about the task, followed by written instructions displayed on the computer screen. The experimental session consisted of the lexical decision task followed by the picture naming task. In both tasks, participants were asked to respond as quickly and accurately as possible.

In the lexical decision task, E-Prime was used for data collection. Reaction times were measured as the interval between the display of the string of letters and the onset of the manual response. In the picture naming task, naming latencies were manually checked with the software Praat version 5.4.08 (Boersma and Weenink, 2015) and were measured as the interval from the presentation of the picture until the speech onset. In both tasks, a trial consisted of (i) a fixation cross for 250 ms, (ii) a blank screen for 250 ms, (iii) the target item (together with the language cue—represented by the background color of the screen—in the picture naming task) for 1,500 ms and (iv) blank screen for 1,000 ms. Independently from subjects' response speed, each trial had a fixed duration of 3,000 ms.

In the lexical decision task, participants were instructed to decide whether a presented string of letters was a real word or not by pressing either a YES or a NO button. Participants responded by using the index finger of their right or left hand to press a button on the right or left side of the keyboard, respectively. The assignment of the button was counterbalanced across participants. The lexical decision task consisted of 336 trials: 1/3 of pseudowords (112 trials) and 2/3 of words (224 trials). Half of the words belonged to the L1 and the other half to the L2. Participants were told that words' language membership was irrelevant for the task.

Items were displayed singularly, but they were organized in pseudo-randomized chunks. There were two types of chunks, namely full and partial word type chunks. The full word type chunks included only words and were composed of 75% repetition and 25% switch trials (total of 252 and 84 trials, respectively). For example, in the chunk L1-L1-L1-L2, the first three members belonged to the same language (L1) and the last one to the other language (L2). Each of the four elements had to be classified as existing words, irrespective of language membership. Only the last two elements of the chunk were included in the analysis (e.g., X-X-L1-L2). We used this system to make sure that every repetition trial was coming from a “pure” repetition trial (note the difference between L2-L1-L1-L2 and L1-L1-L1-L2) and to exclude effects of backwards inhibition on switch trials (note the difference between L1-L2-L1-L2 and L1-L1-L1-L2), for a review on backward inhibition see Koch et al. (2010). Each word was seen only once in each chunk position (i.e., first, second, third and fourth position) of the two language chunks (L1-L1-L1-L2 and L2-L2-L2-L1).

A partial word type chunk entailed both words and pseudowords: the first three components belonged to the same category (word or pseudoword), while the fourth element was in a different category. For example, in a word-word-word-pseudoword chunk, the first three elements had to be classified as existing words and the fourth one as a non-existing word. If the first three components were words, they always belonged to the same language (e.g., L1), while the following pseudoword was generated either from the same language (e.g., L1) or from a different one (e.g., L2). If the first three components of the chunk were pseudowords, they could have been generated from both the L1 and the L2 and the following word was either in the L1 or in the L2. Only the last two elements of the chunk were included in the analysis (e.g., X-X-word-pseudoword).

If two items (e.g., bottle-glasses-X-X) had occurred together in a specific chunk, this items' combination was not repeated twice; additionally, their translations (e.g., fles-bril-X-X) and their derived pseudowords (e.g., boddle-smartes-X-X) were never presented together within a chunk. To avoid orthographic priming, items sharing more similar orthographic patterns (such as the pseudowords derived from the item words) never occurred in the same chunk. Moreover, a given item was never seen within the next 5 trials and the same type of chunk never occurred more than twice in a row. Because of these constraints, the order of the trials was unpredictable. Four lists of the task were created, two starting with a word-word-word-pseudoword and the other two with a pseudoword-pseudoword-pseudoword-word chunk type. Each participant was administered with one list only. The language of instructions for the lexical decision task was Dutch. Before the main experiment, participants were given a practice session of 24 trials. Practice items were not included in the main experiment.

In the picture naming task, participants had to name a presented picture either in their L1 (Dutch) or in their L2 (English). The language to be used was signaled by the background color of the screen (e.g., blue = L1 and green = L2). The assignment of the color cue to the response language was counterbalanced across participants. The picture naming task consisted of a single-language followed by a mixed- language block. In the single-language block, pictures had to be named in either L1 or L2 separately. It included a total of 56 trials: Half of the items had to be named in the L1 and the other half in the L2. For each language, the 14 pictures were randomly presented for two times consecutively. The order of languages' presentation was counterbalanced across participants. The language of instruction corresponded to the language in which the upcoming task had to be performed (English for the upcoming L2 part and Dutch for the following L1 part).

The mixed-language block involved two kinds of trials, repetition and switch trials. In a repetition trial, a given picture had to be named in the same language as in the trial before (e.g., L1-L1). In a switch trial, a presented picture had to be named in a different language compared to the trial before (e.g., L2-L1). The mixed-language block was composed by 112 trials: Half of them had to be named in the L1 and the other half in the L2. Trials were organized in pseudo-randomized language chunks composed of 75% repetition and 25% switch trials (84 and 28 trials, respectively). For example, the language chunk L1-L1-L1-L2 implied that the three consecutive pictures had to be named in the L1 and the fourth one in the L2. For the analysis, only the second part of the language chunk was used (X-X-L1-L2). The same chunk type was never displayed more than two times in a row. Each picture was seen eight times within the mixed-language block, which is once in each position of the language chunk (first, second, third of fourth position) of the two language chunks (L1-L1-L1-L2 and L2-L2-L2-L1). The same picture was not seen within 5 trials. Again the order of the items was unpredictable. Four lists of mixed-language block were created: two starting with a L1-L1-L1-L2 and the other two with a L2-L2-L2-L1 chunk type. Each participant saw only one list. In the mixed language block, the language of instructions was Dutch.

Participants were given one practice sessions before each language part of the single-language block (total of 12 trials) and one practice session before the mixed-language block, in which the two chunk types were trained (total of 16 trials). Practice trials did not appear in the experimental sessions. On average, the experiment (including instructions and breaks) lasted 30 min.

### Data cleaning

The dependent variables of the present study were accuracy rates and reaction times. Before the statistical analyses, we cleaned the dataset based on participants' and items' error rates. Because of low accuracy scores (<66%), one participant and two items were removed from subsequent analyses (pseudowords “hers” and “croud” of the lexical decision task). Accuracy rates for all other participants and items ranged, respectively from 73 to 100% and from 79 to 100%.

Both correct and incorrect responses were included in the accuracy rates' analyses, whereas only correct responses were used to analyse reaction times. We deemed a response as incorrect in the case of wrong button pressing for the lexical decision task (4.5% of the data) and in the case of microphone miss-triggering (e.g., coughing, hesitation, utterance repairs, etc.) and selection of the wrong word and/or language for the picture naming task (9.8% of the data). In both tasks, missing responses were classified as incorrect.

After exclusion of the incorrect responses, we screened reaction times for extreme values. Extremely fast (<150 ms) or particularly slow (> 2,500 ms) reaction times were removed from the dataset (0.03% of the data). All the analyses were carried out in GNU-R version 3.2.2 (R Core Team, 2015) using the lme4 package version 1.1-9 (Bates et al., 2015). Reaction times data were fitted in linear mixed effects models, while accuracy binary data (1 = correct, 0 = incorrect) were fitted in generalized linear mixed effects models with a logistic link function. For detailed information on data analysis see Appendix C in Supplementary Material.

## Results

To investigate the assumptions outlined in the introduction, we measured L1 and L2 switching costs (calculated as the RT difference between repetition and switch trials) in the lexical decision and in the picture naming tasks. Consider first the lexical decision task. Mean accuracy rates and reaction times are illustrated in Table 1. Results of the statistical analysis for the accuracy rate and the reaction time data are reported in Tables 2, 3, respectively.

**Table 1:** Correct mean reaction times in milliseconds (standard deviation in brackets) and accuracy rates in percent for Words and Pseudowords as a function of Response repetition (yes vs. no), Language (L1 vs. L2) and Condition (Repetition vs. Switch).

		**Response repetition (yes)**			**Response repetition (no)**		
		**L1**	**L2**	**Mean**	**L1**	**L2**	**Mean**
**(A) WORD**							
Repetition	*Reaction times*	472 ms (126)	516 ms (170)	494 ms (151)	590 ms (173)	587 ms (147)	589 ms (160)
	*Accuracy rates*	100% (5)	98% (12)	99% (9)	97% (17)	93% (24)	95% (21)
Switch	*Reaction times*	507 ms (143)	514 ms (139)	511 ms (141)	564 ms (130)	587 ms (119)	574 ms (125)
	*Accuracy rates*	98% (12)	96% (18)	97% (16)	97% (17)	93% (26)	95% (22)
SWITCHING COSTS		*35 ms*	*−2 ms*		*−26 ms*	*0 ms*	
Language Mean	*Reaction times*	484 ms (133)	515 ms (160)	500 ms (148)	576 ms (152)	587 ms (133)	581 ms (143)
	*Accuracy rates*	99% (8)	98% (15)	98% (12)	97% (17)	93% (25)	95% (21)
**(B) PSEUDOWORD**							
Repetition	*Reaction times*	627 ms (177)	635 ms (165)	631 ms (171)	617 ms (122)	621 ms (153)	619 ms (139)
	*Accuracy rates*	98% (14)	99% (10)	98% (12)	85% (36)	90% (31)	87% (33)
Switch	*Reaction times*	640 ms (188)	637 ms (209)	638 ms (199)	590 ms (92)	604 ms (93)	596 ms (91)
	*Accuracy rates*	95% (21)	95% (22)	95% (22)	94% (23)	95% (21)	95% (22)
SWITCHING COSTS		*13 ms*	*2 ms*		*−27 ms*	*−17 ms*	
Language Mean	*Reaction times*	634 ms (183)	636 ms (188)	635 ms (186)	612 ms (117)	619 ms (148)	616 ms (133)
	*Accuracy rates*	96% (18)	97% (18)	97% (18)	87% (34)	90% (30)	88% (32)

*Language membership in Pseudowords indicates whether they were generated from a L1 or a L2 word. Switching costs (calculated as the difference between repetition and switch trials) are reported in italics*.

**Table 2:** Estimated coefficients, standard errors (SE) and *z*-values from the best-fit generalized linear mixed-effects models for the accuracy data.

	**Accuracy rates**		
	**Estimate**	**SE**	***z*-value**
**(A) OVERALL MODEL**			
Intercept	3.64	0.20	18.18^****^
Language (L1 vs. L2)	0.19	0.10	1.80
Condition (Repetition vs. Switch)	0.18	0.10	1.76
Category (Word vs. Pseudoword)	0.35	0.10	3.35^***^
Response repetition (yes vs. no)	0.61	0.10	5.79^****^
Language^*^Condition	−0.04	0.10	−0.43
Language^*^Category	0.32	0.10	3.07^**^
Condition^*^Category	0.14	0.10	1.30
Language^*^Response repetition	0.03	0.10	0.33
Condition^*^Response repetition	0.41	0.10	3.84^***^
Category^*^Response repetition	0.01	0.10	0.09
Language^*^Condition^*^Category	0.10	0.10	1.03
Language^*^Condition^*^Response repetition	0.02	0.10	0.25
Language^*^Category^*^Response repetition	0.04	0.10	0.45
Condition^*^Category^*^Response repetition	−0.14	0.10	−1.34
Language^*^Condition^*^Category^*^Response repetition	0.07	0.10	0.69
Formula: Accuracy ~ Language^*^Condition^*^Category^*^Response repetition + (1|subject) + (1|item)			
**(B) WORD**			
Intercept	4.36	0.28	15.40^****^
Language (L1 vs. L2)	0.45	0.22	2.03^*^
*Formula: Accuracy ~ Language + (Language|subject) + (1|item)*			
**(C) PSEUDOWORD**			
Intercept	2.80	0.19	14.47^****^
Language (L1 vs. L2)	−0.14	0.09	−1.47
*Formula: Accuracy ~ Language + (1|subject) + (1|item)*			
**(D) REPETITION TRIALS**			
Intercept	3.67	0.21	17.05^****^
Response repetition (yes vs. no)	1.18	0.11	10.14^****^
*Formula: Accuracy ~ Response repetition + (1|subject) + (1|item)*			
**(E) SWITCH TRIALS**			
Intercept	3.45	0.24	14.37^****^
Response repetition (yes vs. no)	0.22	0.12	1.78
*Formula: Accuracy ~ Response repetition + (1| subject) + (1|item)*			

Asterisks (^*^) indicate:

* *p < 0.05*,

** *p < 0.01*,

*** *p < 0.001*,

and

**** *p < 0.0001*.

**Table 3:** Estimated coefficients, standard errors (SE), and *t*-values from the best-fit linear mixed effects models run on reciprocal square root-transformed RTs.

	**Reaction times**		
	**Estimate**	**SE**	***t*-value**
**(A) OVERALL MODEL**			
Intercept	−42.57	0.41	−103.11^****^
Language (L1 vs. L2)	−0.23	0.17	−1.37
Condition (Repetition vs. Switch)	0.06	0.08	0.70
Category (Word vs. Pseudoword)	−1.59	0.17	−9.21^****^
Response repetition (yes vs. no)	−0.82	0.14	−5.63^****^
Language^*^Condition	−0.14	0.08	−1.71
Language^*^Category	−0.20	0.07	−2.64^*^
Condition^*^Category	−0.17	0.08	−2.09^*^
Language^*^Response repetition	−0.03	0.07	−0.42
Condition^*^Response repetition	−0.19	0.09	−2.18^*^
Category^*^Response repetition	−0.92	0.07	−12.24^****^
Language^*^Condition^*^Category	0.06	0.08	0.84
Language^*^Condition^*^Response repetition	−0.22	0.08	−2.58^*^
Language^*^Category^*^Response repetition	−0.06	0.07	−0.90
Condition^*^Category^*^Response repetition	−0.23	0.08	−2.65^*^
Language^*^Condition^*^Category^*^Response repetition	−0.02	0.08	−0.31
Formula: RT ~ Language^*^Condition^*^Category^*^Response repetition + (Language+Category+Response repetition|subject) + (Language+Category+Response repetition| item)			
**(B) WORD–OVERALL MODEL**			
Intercept	−44.13	0.43	−102.45^****^
Language (L1 vs. L2)	0.91	0.16	5.72^****^
Condition (Repetition vs. Switch)	0.35	0.16	2.12^*^
Response repetition (yes vs. no)	3.54	0.27	12.98^****^
Language^*^Condition	−0.37	0.33	−1.13
Language^*^Response repetition	−0.46	0.32	−1.45
Condition^*^Response repetition	−1.39	0.34	−4.15^****^
Language^*^Condition^*^Response repetition	1.58	0.66	2.39^*^
Formula: RT ~ Language^*^Condition^*^ Response repetition + (Response repetition|subject) + (1|item)			
**(C) WORD–*****RESPONSE REPETITION (YES)***			
Intercept	−45.86	0.44	−102.53^****^
Language (L1 vs. L2)	1.09	0.22	4.95^****^
Condition (Repetition vs. Switch)	1.05	0.17	6.04^****^
Language^*^Condition	−1.25	0.34	−3.59^***^
Formula: RT ~ Language^*^Condition + (Language|subject) + (Language^*^Condition|item)			
**(D) WORD–*****RESPONSE REPETITION (YES) L1***			
Intercept	−46.46	0.50	−91.55^****^
Condition (Repetition vs. Switch)	1.66	0.37	4.41^****^
*Formula: RT ~ Condition + (1|subject) + (Condition|item)*			
**(E) WORD–*****RESPONSE REPETITION (YES) L2***			
Intercept	−45.39	0.52	−86.37^****^
Condition (Repetition vs. Switch)	0.29	0.53	0.55
*Formula: RT ~ Condition + (1|subject) + (Condition|item)*			
**(F) WORD–*****RESPONSE REPETITION (NO)***			
Intercept	−42.49	0.46	−91.08^****^
Language (L1 vs. L2)	0.63	0.22	2.85^**^
Condition (Repetition vs. Switch)	−0.46	0.31	−1.45
Language^*^Condition	0.02	0.54	0.04
Formula: RT ~ Language^*^Condition + (Condition|subject) + (1|item)			
**(G) PSEUDOWORD–OVERALL MODEL**			
Intercept	−40.90	0.46	−87.26^****^
Language (L1 vs. L2)	−0.17	0.29	−0.58
Condition (Repetition vs. Switch)	0.18	0.16	1.16
Response repetition (yes vs. no)	0.11	0.15	0.72
Language^*^Condition	−0.11	0.15	−0.73
Language^*^Response repetition	0.01	0.20	0.09
Condition^*^Response repetition	0.15	0.18	0.87
Language^*^Condition^*^Response repetition	−0.27	0.17	−1.58
Formula: RT ~ Language^*^Condition^*^Response repetition + (Language^*^Response repetition|subject) + (Language^*^Response repetition|item)			

Asterisks (^*^) indicate:

* *p < 0.05*,

** *p < 0.01*,

*** *p < 0.001*,

and

**** *p < 0.0001*.

### Lexical decision task

The analysis of the accuracy rates revealed that responses were significantly more accurate in words than in pseudowords (β = 0.35, *SE* = 0.10, *z* = 3.35, *p* < 0.01). A response change (e.g., from pseudoword to word) yielded significantly less accurate responses compared to when the same response was repeated (β = 0.61, *SE* = 0.10, *z* = 5.79, *p* < 0.0001). The significant interaction of Language by Category (β = 0.32, *SE* = 0.10, *z* = 3.07, *p* < 0.01), indicated that compared to pseudowords, words were responded more accurately in the L1 than in the L2. Specifically, while accuracy rates did not differ significantly for pseudowords generated from the L1 and those generated from the L2 (*p* > 0.05), L1 words were responded more accurately than L2 words (β = 0.45, *SE* = 0.28, *z* = 2.03, *p* < 0.05). Finally, we found a significant interaction between Condition and Response repetition (β = 0.41, *SE* = 0.10, *z* = 3.84, *p* < 0.001), indexing that a change of response affected language repetition and switch trials differently. In particular, compared to a situation in which the same response is repeated (e.g., word-word), a change of response (e.g., pseudoword-word) yielded significantly lower accuracy rates for repetition trials (β = 1.18, *SE* = 0.11, *z* = 10.14, *p* < 0.0001), but not so for switch trials (*p* > 0.05).

As expected, the analysis of the reaction times showed that pseudowords were responded significantly slower than words (β = 1.59, *SE* = 0.17, *t* = 9.21, *p* < 0.0001) and that response change yielded slower responses compared to response repetition (β = 0.82, *SE* = 0.14, *t* = 5.63, *p* < 0.0001). The main effects of Language and Condition were not significant (*p* > 0.05). However, the significant interactions of Language by Category (β = 0.20, *SE* = 0.07, *t* = 2.64, *p* < 0.05) and of Condition by Category (β = 0.17, *SE* = 0.08, *t* = 2.09, *p* < 0.05) revealed that compared to pseudowords, words were responded faster in the L1 than in the L2 and in repetition compared to switch trials. Both the interactions of Category by Response repetition and of Condition by Response repetition were also significant (β = 0.92, *SE* = 0.07, *t* = 12.24, *p* < 0.0001 and β = 0.19, *SE* = 0.09, *t* = 2.18, *p* < 0.05), indicating that a response change was more costly for repetition compared to switch trials and for words than for pseudowords. Both the three-way interactions between Language, Condition and Category and between Condition, Category and Response repetition were significant (β = 0.22, *SE* = 0.08, *t* = 2.58, *p* < 0.05 and β = 0.23, *SE* = 0.08, *t* = 2.65, *p* < 0.05, respectively). All the other interactions were not significant (*p* > 0.05).

To investigate these interactions, we split the data into words and pseudowords. With regard to words, we found that responses were faster in the L1 than in the L2 (β = 0.91, *SE* = 0.16, *t* = 5.72, *p* < 0.0001) and in repetition compared to switch trials (β = 0.35, *SE* = 0.16, *t* = 2.12, *p* < 0.05). The effect of Response repetition was also significant (β = 3.54, *SE* = 0.27, *t* = 12.98, *p* < 0.0001), indicating that responses were significantly slower if the preceding trial was a pseudoword compared to a word. This effect was smaller for switch compared to repetition trials (β = 1.39, *SE* = 0.34, *t* = 4.15, *p* < 0.001). The three-way interaction between Language, Condition and Response repetition was also significant (β = 1.58, *SE* = 0.66, *t* = 2.39, *p* < 0.05). No other interaction was significant (*p* > 0.05). Words were further divided based on the category of the preceding trial (Response repetition: yes vs. no).

When the same response was repeated (i.e., Response repetition: yes), words were responded faster in the L1 relative to L2 (β = 0.54, *SE* = 0.11, *t* = 4.95, *p* < 0.0001) and in repetition compared to switch trials (β = 0.52, *SE* = 0.08, *t* = 6.04, *p* < 0.0001). The difference between repetition and switch trials was larger for L1 compared to L2 words (β = 0.31, *SE* = 0.08, *t* = 3.59, *p* < 0.001), that is *asymmetrical switching costs*. In particular, the effect of language switching was significant for the L1 (β = 0.83, *SE* = 0.18, *t* = 4.41, *p* < 0.001), but not for the L2 (*p* > 0.05). With reference to words preceded by pseudowords (i.e., Response repetition: no), responses were faster in the L1 compared to the L2 (β = 0.63, *SE* = 022, *t* = 2.85, *p* < 0.01). No other main effect or interaction was significant (*p* > 0.05) in this condition. Concerning pseudowords, items generated from the L1 were responded equally fast as those generated from the L2 (*p* < 0.05). All the other main effects and interactions were also not significant (*p* > 0.05).

To sum up, performances were more accurate and faster for words than for pseudowords and in the case of response repetition (e.g., word-word) compared to response change (e.g., pseudoword-word). Changing response was particularly costly for words and repetition trials than for pseudowords and switch trials, respectively. The effect of Language was influential for words, in that L1 words were responded faster and more accurately than L2 words, but not for pseudowords. The effect of Condition also affected words only, with responses on switch trials being slower and less accurate than on repetition trials. This difference was significant for the L1 and not significant for the L2, i.e., asymmetrical switching costs.

### Picture naming task

Consider now the picture naming task. Mean accuracy rates and reaction times are presented in Table 4. Results of the statistical analysis for the accuracy rate and the reaction time data are reported in Tables 5, 6, respectively.

**Table 4:** Mean reaction times in milliseconds (standard deviations in brackets) and accuracy rates in percent for correct responses of L1 vs. L2 in single-language block (upper part) vs. mixed-language block (lower part).

		**L1**	**L2**	**Mean**
**SINGLE-LANGUAGE BLOCK**				
Mean	*Reaction times*	704 ms (175)	729 ms (173)	716 ms (174)
	*Accuracy rates*	92% (27)	90% (30)	91% (28)
**MIXED-LANGUAGE BLOCK**				
Repetition	*Reaction times*	773 ms (179)	744 ms (179)	758 ms (171)
	*Accuracy rates*	90% (30)	95% (22)	93% (26)
MIXING COSTS		*69 ms*	*15 ms*	
Switch	*Reaction times*	819 ms (162)	797 ms (175)	808 ms (177)
	*Accuracy rates*	89% (31)	89% (31)	89% (31)
SWITCHING COSTS		*46 ms*	*53 ms*	
Mean	*Reaction times*	796 ms (180)	770 ms (171)	783 ms (176)
	*Accuracy rates*	90% (31)	92% (27)	91% (29)

*For the mixed-language block repetition vs. switch trials are reported. Mixing costs (calculated as the difference between trials in the single-language block and repetition trials in the mixed-language block) and switching costs (calculated as the difference between repetition and switch trials) and for the L1 and the L2 are reported in italics*.

**Table 5:** Estimated coefficients, standard errors (SE) and *z*-values from the best-fit generalized linear mixed-effects models for the accuracy data.

	**Reaction times**		
	**Estimate**	**SE**	***z*-value**
**(A) SINGLE-LANGUAGE BLOCK**			
Intercept	2.66	0.19	13.34^****^
Language (L1 vs. L2)	0.07	0.15	0.46
*Formula: Accuracy ~ Language + (Language|subject) + (Language|item)*			
**(B) MIXED-LANGUAGE BLOCK**			
Intercept	2.93	0.25	11.71^****^
Language (L1 vs. L2)	−0.34	0.13	−2.54^*^
Condition (Repetition vs. Switch)	−0.50	0.17	−2.91^**^
Language^*^Condition	0.34	0.17	1.97
Formula: Accuracy ~ Language^*^Condition + (1|subject) + (Language|item)			
**(C) MIXING COSTS–OVERALL MODEL**			
Intercept	2.798	0.198	14.09^****^
Language (L1 vs. L2)	−0.206	0.135	−1.49
Block (Single vs. Mixed)	−0.150	0.088	−1.83
Language^*^Block	0.257	0.082	3.13^**^
Formula: Accuracy ~ Language^*^Block + (Language|subject) + (1|item			
**(D) MIXING COSTS–L1**			
Intercept	2.603	0.211	12.29^****^
Block (Single vs. Mixed)	0.111	0.103	1.07
*Formula: Accuracy ~ Block + (1|subject) + (1|item)*			
**(E) MIXING COSTS–L2**			
Intercept	3.031	0.283	10.70^****^
Block (Single vs. Mixed)	−0.41	0.128	−3.26^**^
*Formula: RT ~ Block + (1 ±*subject*)+(1±*item*)*			

Asterisks (^*^) indicate:

* *p < 0.05*,

** p < 0.01

and ^***^ p < 0.001.

**Table 6:** Estimated coefficients, standard errors (SE) and *t*-values from the best-fit linear mixed effects models run on log-transformed RTs.

	**Reaction times**		
	**Estimate**	**SE**	***t*-value**
**(A) SINGLE-LANGUAGE BLOCK**			
Intercept	6.54	0.02	271.77^****^
Language (L1 vs. L2)	−0.01	0.01	−0.96
*Formula: RT ~ Language + (Language|subject) + (1|item)*			
**(B) MIXED-LANGUAGE BLOCK**			
Intercept	6.684	0.023	279.60^****^
Language (L1 vs. L2)	−0.033	0.008	−3.99^***^
Condition (Repetition vs. Switch)	0.064	0.017	3.67^***^
Language^*^Condition	0.006	0.016	0.39
Formula: RT ~ Language^*^Condition + (1+ Condition|subject) + (Condition|item)			
**(C) MIXING COSTS–OVERALL MODEL**			
Intercept	6.576	0.024	273.52^****^
Language (L1 vs. L2)	0.001	0.012	0.10
Block (Single vs. Mixed)	−0.028	0.010	−2.70^*^
Language^*^Block	−0.017	0.006	−2.85^**^
Formula: RT ~ Language^*^Block + (Language^*^Block|subject) + (Language^*^Block|item)			
**(D) MIXING COSTS–L1**			
Intercept	6.578	0.028	231.56^****^
Block (Single vs. Mixed)	−0.048	0.011	−4.34^***^
*Formula: RT ~ Block + (1|subject) + (Block|item)*			
**(E) MIXING COSTS–L2**			
Intercept	6.574	0.025	254.95^****^
Block (Single vs. Mixed)	−0.010	0.009	−1.09
*Formula: RT ~ Block + (Block|subject) + (1|item)*			

The analysis of the accuracy rates showed that in the single-language block responses in L1 and L2 were equally accurate (*p* > 0.05). In the mixed-language block, responses were significantly more accurate in the L2 than in the L1 (β = 0.34, *SE* = 0.13, *z* = 2.54, *p* < 0.05) and in repetition compared to switch trials (β = 0.50, *SE* = 0.17, *z* = 2.91, *p* < 0.05). The marginal significant interaction of Language by Condition (β = 0.34, *SE* = 0.17, *z* = 1.97, *p* = 0.06) indicates that compared to repetition trials, switch trials tended to be less accurate in the L1 than in the L2. The accuracy rates' difference between the single- and the mixed-language blocks was not significant (*p* > 0.05). However, there was a significant interaction between Language and Block (β = 0.25, *SE* = 0.08, *z* = 3.13, *p* < 0.01), indicating that compared to the single-language block, mixed-language block responses were more accurate for the L2 than for the L1. Specifically, while the L2 was responded to significantly better in the mixed- compared to the single-language block (β = 0.41, *SE* = 0.12, *z* = 3.26, *p* < 0.01), L1 accuracy rates was not influenced by the type of block (*p* > 0.05).

The analysis of the reaction times revealed that there was no significant difference between L1 and L2 responses in the single-language block (*p* > 0.05) and that in the mixed-language block, responses were faster in the L2 than in the L1 (β = 0.03, *SE* = 0.008, *t* = 3.99, *p* < 0.001). Overall, participants responded slower on repetition trials of the mixed-language block compared to trials in the single-language block (β = 0.28, *SE* = 0.10, *t* = 2.70, *p* < 0.05), i.e., *mixing costs*. This difference was greater for the L1 than for the L2, as indicated by the significant interaction of Language and Block (β = 0.017, *SE* = 0.006, *t* = 2.85, *p*< 0.05). In particular, while L1 responses were significantly slower in the mixed- compared to the single-language block (β = 0.04, *SE* = 0.01, *t* = 4.34, *p* < 0.001), L2 responses were not affected by the type of block (*p* > 0.05). In the mixed language block, switch trials were responded to slower than repetition trials (β = 0.06, *SE* = 0.01, *t* = 3.67, *p* < 0.001), and this effect was comparable for the L1 and the L2 (*p* > 0.05), which means that we found *symmetrical switching costs*. However, given the relatively small number of trials used in this task, we performed an additional analysis in order to assess whether the observed data might have suffered from unwanted biases. To do so, we used Bayesian hypothesis testing that allows for rigorous quantification of statistical evidence (Wagenmakers, 2007) and it is supposed to be appropriate to deal with the uncertainty of small samples (Hinneburg et al., 2007). More precisely, we calculated the Bayes factor, which is considered a way of evaluating evidence in favor of the null or the alternative hypothesis, given the observed data (Jeffreys, 1935). A Bayes factor smaller than 1 indicates that the observed data are more likely under the alternative hypothesis (H1) than under the null hypothesis (H0). With concern to the picture naming task, we formulated the null hypothesis as predicting a comparable effect of language switching between the two languages (symmetrical switching costs) and the alternative hypothesis as expecting a different effect of language switching on the two languages (asymmetrical switching costs). To calculate the Bayes factor we compared the Schwarz criterion (or BIC) information of the H0 model (symmetrical switching costs) and the H1 model (asymmetrical switching costs). Results showed a Bayes factor of 36.41, indicating strong evidence (88% probability) in favor of the null hypothesis (for details on the Bayes factor calculation and interpretation see Wagenmakers, 2007). Thus, the Bayesian inference indicates that there is strong evidence that language switching costs for the two languages is symmetrical in the production task. For the sake of completeness, we calculated the Bayes factor also in relation to the language switching results of the lexical decision task. In this case, the comparison between the BIC information of the H0 (symmetrical switching costs) and the H1 (asymmetrical switching costs) yielded a Bayes factor of.004, indicating that the data are more likely (99% probability) under the H1 than the H0. Hence, the Bayesian inference indicates that there is a very strong evidence in favor of the fact that language switching costs in the lexical decision task are asymmetrical.

To summarize the results of the picture naming task, both accuracy rates and reaction time analyses showed that in the single-language block there was no difference between L1 and L2. In the mixed-language block, responses were more accurate and faster in the L2 compared to the L1 and in repetition than in switch trials. Responses in the L1 were equally accurate in repetition trials of the mixed-language block compared to trials of the single-language block and were responded to slower in the former than in the latter, which means that there were mixing costs. Responses in the L2 were more accurate in repetition trials of the mixed-language block than in trials of the single-language block and did not suffer from mixing costs. Finally, within the mixed-language block, the difference between repetition and switch trials was similar for the L1 and the L2, indexing symmetrical switching costs.

## Discussion

The goal of the study was to investigate language control in bilingual recognition and production. More precisely, we aimed at assessing whether (1) language inhibition in recognition is dominance-related (as the IC predicts) or dominance-reversed (as the BIA predicts) and from this at investigating whether (2) language control in bilingual recognition and production rely on the same or different mechanisms. To address these issues, we measured language switching costs in a group of native speakers of Dutch (L1)-proficient learners of English (L2) performing a bilingual lexical decision and a bilingual picture naming task.

### Lexical decision task

#### Language switching costs

We regard to the main research question, we found that language switching costs for words were larger for the L1 compared to the L2, i.e., asymmetrical switching costs. More precisely, responses were significantly slower on switch than on repetition trials for the L1, but not for the L2 where reaction times in switch and repetition trials were comparable. This result replicates Jackson et al. (2004) study, where unbalanced bilinguals (L1 English–different L2s) showed language switching cost only in the L1 but not in the L2 while performing a parity judgment task (i.e., classifying a digit as odd or even).

Why did we find language switching costly for the L1, but not for the L2? Before answering this question, it is important to understand the role of language strength of activation in word recognition. As proposed by the BIA model the speed with which a word is recognized depends on its baseline activation level, with more frequent words (such as L1 words) being recognized faster than less frequent words (such as L2 words). Additionally, the speed with which a word is recognized also depends on its relation to other words in the lexicon (e.g., Dijkstra and van Heuven, 1998). That being said, in order to explain the results obtained in the present study, we further assume that when a word from a weaker L2 is presented (e.g., the L2 word “BOTTON”), competing words from both the L2 and L1 will be activated. The activated words will send activation to the corresponding language node. In our example, the L2 word “BOTTON” will excite the L2 language node more than the L1 language node. The activated language nodes will inhibit words belonging to the competing language so that L1 words will be strongly inhibited by the L2 language node. Therefore, when on a switch trials, an L1 word is presented, this needs to overcome the previously applied inhibition before being recognized, i.e., L1 switching costs. However, when a word from the stronger L1 is presented (e.g., the Dutch word “KNOP”), mostly words from the L1 will activate and act as competitors, but not so many words from the L2. This is because L1 words have a higher baseline activation level than L2 words, and therefore when an L1 word is presented, L1 candidates are activated faster and more strongly than L2 ones. Therefore, if when L1 words are presented, competition from the L2 words is relatively weak, then the inhibition applied to the non-relevant L2 will also be relatively weak. Consequently, when on a switch trial the L2 becomes relevant again, the cost to reactivate this language will be small or absent, i.e., undetectable L2 switching costs.

This assumption has two main implications. Firstly, it assumes that the amount of inhibition exerted on words of the non-relevant language increases along with their activation level (more strongly activated words need to be inhibited more). This assumption is line with the IC model predictions, according to which the magnitude of inhibition depends on languages' activation strength. Yet, this is in contrast with the BIA model proposal that the L1 language node inhibits L2 words to a greater extent than vice versa (e.g., Dijkstra and van Heuven, 1998; van Heuven et al., 1998), and with the idea that, because of their higher resting level, L1 words interfere more when switching into the L2 than the other way around, yielding larger switching costs for the L2 than for the L1 (Grainger et al., 2010). Secondly, it suggests that not only similar but also dissimilar words are activated and compete for selection. Specifically, despite the fact that no cognate, homograph or neighbor words were included in the present experiment, words from the non-relevant language were activated as indicated by the language switching costs measured in L1. Finally, asymmetrical switching costs in advanced L2 speakers challenge the hypothesis suggesting that when the dominance difference between a stronger and a weaker language is relatively small, the amount of inhibition applied to the two languages is comparable, leading to symmetrical switching costs.

As described earlier, we found no significant switching costs for the L2. This result is in contrast with previous findings on bilingual lexical decision studies, in which the same amount of switching costs was found in L1 and L2 (e.g., Thomas and Allport, 2000; Orfanidou and Sumner, 2005). The main difference between the present study and previous studies lies in the type of stimuli used. While the present study included only items with language-nonspecific orthography, previous studies entailed items with language-specific and language-nonspecific orthography. When items with language–specific orthography are used in the task, both words from the L1 and the L2 are recognized faster compared to a situation when items have language-nonspecific orthography (Thomas and Allport, 2000; Orfanidou and Sumner, 2005). This effect seems to be greater for the weaker L2 than for the stronger L1 (e.g., L2 words benefi*t* = 91 ms and L1 words benefit = 26 ms, in Thomas and Allport, 2000) and could be explained by the fact that language specific orthography is more helpful in a more complex situation (e.g., during L2 word recognition) than when the system is already relatively fast in recognizing the appropriate word (e.g., during L1 word recognition). Therefore, in the case of language-specific orthography, for both L1 and L2 activation is mostly send to words of the relevant language, while words from the irrelevant language will be coactivated very weakly. In support of this idea, Orfanidou and Sumner (2005) showed that when only items with a language-specific orthography are used within a block, language switching costs are extremely reduced. However, the authors found that when language-specific and language-nonspecific stimuli are intermingled in the same block, the amount of switching costs for language-specific increases to the point that switching costs for language-specific and language-nonspecific stimuli become comparable. Moreover, replicating Thomas and Allport (2000) results, the authors did not find any significant interaction of language and switching costs, indicating overall symmetrical switching costs. Unfortunately, none of the two above-mentioned studies reported whether language switching pattern was modulated by orthography specificity (namely, no information was provided on the three-way interaction of language, switching costs, and orthography specificity). This makes it difficult to compare results from those studies, in which language-specific and language-nonspecific stimuli were intermingled in the same experiment with the findings of the present study, which included only language-nonspecific stimuli. The present study is also difficult to compare with von Studnitz and Green (1997) study, despite the fact that, like in the present study, only stimuli with language-nonspecific orthography were used there. In their study, authors tested native speakers of German (L1) - highly proficient speakers of English (L2) in a bilingual lexical decision study. At the time of testing, all participants were living in the L2 environment and reported being predominantly exposed to their L2 than to their L1. Therefore, it is possible that L2 lexical representation in this group of participants is not comparable to that of the participants of the present study. The same reasoning holds for Grainger and Beauvillain (1987) investigating bilingual word recognition in English (L1)–French (L2) speakers, who were pupils of bilingual schools in Paris and, therefore, equally exposed to the two languages.

#### Pseudowords

In the lexical decision task, participants responded faster and more accurately to words than to pseudowords replicating the well-established facilitatory effect of words over pseudowords (e.g., Meyer and Schvaneveldt, 1971; Weekes, 1997; MacGregor et al., 2012). Even though pseudowords were generated starting from existing L1 and L2 words, the effect language did not influence participants' performances. This indicates that overall pseudowords were properly matched, in that it was no longer possible to quickly associate a given pseudoword to a specific language. However, a closer inspection of the data revealed that there was a tendency for pseudowords generated from the L1 to be more error prone than the ones generated from the L2. As noted in previous studies, responses on pseudowords with many neighbors are hampered to a greater extent compared to pseudowords with fewer neighbors (Coltheart et al., 1977; Balota et al., 2004). To avoid this effect, pseudowords of the present study were matched according to their orthographic Levenshtein distance to real words in the lexicon. However, it might be possible that if a speaker is more dominant in one language (L1) than in another (L2), pseudowords generated from a more dominant language will coactivate a larger number of neighbors, than pseudowords generated from a less dominant language. This imbalance in the speakers' languages dominance could explain why pseudowords created from the L1 tended to be less accurate than pseudowords generated from the L2.

As expected, the difference between repetition and switch trials was not significant for pseudowords. The absence of language switching costs for pseudowords replicates previous studies on bilingual language control using a lexical decision task (Thomas and Allport, 2000, Exp. 1; von Studnitz and Green, 1997, Exp. 2). However, preceding studies have also shown that pseudowords can show language switching costs. Language switching costs for pseudowords were found when on a trial involving language switch the pseudowords had orthographic and phonological characteristics of the preceding language (Thomas and Allport, 2000, Exp. 2), when pseudowords were legal strings only in the current language (von Studnitz and Green, 1997, Exp. 1), but also when pseudowords shared lexical properties with both languages (von Studnitz and Green, 1997, Exp. 1; Orfanidou and Sumner, 2005). While language switching costs for pseudowords with properties of a specific language might be explained by the fact that those specific properties make it possible to associate a pseudoword with a determined language, it is less clear why pseudowords that are legal strings in both languages can generate language switching costs. The only plausible explanation for this is that in previous studies pseudowords were not successfully matched and participants were still able to associate a given pseudoword with a specific language. For that reason, it was important to make sure that the pseudowords generated in the present study were language neutral and could not be easily attributed to either language.

#### Change of response

A change in response was costly only for words, but not for pseudoword. This means that words were responded slower and less accurately if preceded by pseudowords and that, pseudowords performances were not affected by the response type of the preceding trial. This effect might be explained by the fact that in a lexical decision task, participants are faced with two tasks of unequal difficulty, as recognizing pseudowords is more costly than recognizing real words. Specifically, it might be the case that the effort of recognizing pseudowords has a carry-over effect on the next trial. This carry-over effect on upcoming trials is detectable in the case of relatively fast trials, such as word recognizing, while it takes more time to dissipate in the case of relatively slower trials, such as pseudowords recognition. It should be noted that in previous monolingual lexical decision tasks, both words and pseudowords were found to be responded to slower if the preceding trial was a pseudoword compared to a word (Lima and Huntsman, 1997; Perea and Carreiras, 2003). However, the difference between previous monolingual studies and the present study might be explained by the fact that pseudowords created from a single language are faster to recognize than pseudowords sharing orthographic rules with two languages. Therefore, the potential carry-over effect from the preceding trial is still detectable when pseudowords are relatively easier to recognize (monolingual task), but less so when pseudowords are more difficult to identify (bilingual task). In this respect, it is interesting to note that, in the present study, there is a numerical trend for pseudowords preceded by pseudowords to be slower than pseudowords preceded by words.

As to language switching costs, we found that language switching was costly only when words were preceded by words, but not when words were preceded by pseudowords. The absence of language switching costs in the case of response change (pseudowords-words) replicates previous studies on bilingual recognition (Thomas and Allport, 2000; von Studnitz and Green, 2002; Orfanidou and Sumner, 2005). Interestingly, according to previous studies, the effects of response switch can be informative on the mechanisms underpinning language control. For example, according to Thomas and Allport (2000), the result that language switching is costly only when the same response is repeated, but not when the response is changed, suggests that language switching costs origin outside the bilingual lexicon (i.e., they are a feature of the response mechanism) and not inside the lexicon. A similar view is held by Orfanidou and Sumner (2005) according to which the fact that language switching costs arise only when the response is repeated and that this effect is not influenced by the orthographic properties of the stimuli indicates that a portion of language switching costs origins outside the recognition system. Likewise, von Studnitz and Green (2002) argued that if switching costs are visible only in the case of response repetition, such costs are not caused by word recognition but by the mapping of decisions into responses. Moreover, according to von Studnitz and Green (2002), this effect can be explained by the fact that a language change might unconsciously boost a change in response and, therefore, lead to faster responses when it overlaps with response change compared to when the same response is repeated. However, if this explanation is correct, then it implies that pseudowords' language membership is still detectable and that it affects speakers' responses on upcoming trials. Alternatively, we suppose that in the case of response change, language switching is not costly because pseudowords do not belong to any language and, therefore, cannot lead to language switching costs on upcoming words. Therefore, whilst we believe that response change is an influential variable within lexical decision tasks, which needs to be taken into consideration, we are skeptical that this variable can provide helpful information about the specific mechanisms supporting language control.

### Picture naming task

#### Language switching costs

With regard to language switching costs, we found that in the mixed-language block, repetition trials were named faster than switch trials and that this effect was the same for the L1 and the L2, i.e., symmetrical switching costs. Symmetrical switching cost for the L1 and the L2 of highly proficient bilinguals have been extensively reported in the language switching literature (Costa and Santesteban, 2004; Costa et al., 2006; Declerck et al., 2013; Fink and Goldrick, 2015). Within a less conservative dominance-related inhibitory account, it has been suggested that when the difference between L1 and L2 dominance is small (such in the case of highly proficient L2 speakers), then the amount of reactive inhibition applied to the two languages when they are non-relevant is similar, leading to symmetrical reactivation costs. With less conservative, it is meant that the relationship between language dominance and the amount of inhibition on that language is less “tight” compared to a more conservative inhibitory account, where language dominance and the amount of inhibition on that language should be strictly related. Hence, in a less conservative account, asymmetrical switching costs are predicted only in the cases of substantial dominance difference between two languages (e.g., in low proficient L2 speakers), while in a more conservative inhibitory account, any degree of dominance difference between two languages should lead to asymmetrical switching costs. Consequently, symmetrical switching costs between a stronger and a weaker language cannot be accounted for by a more conservative view of the IC model, according to which language switching costs are dominance-related (the stronger the language the larger the cost). In such a case, we should have found larger costs for the stronger L1 than for the weaker L2.

It could be argued that the symmetrical switching costs found in the production, but not in the recognition task, might be ascribed to the order in which the two tasks were presented (i.e., the lexical decision task preceded the picture naming task). In particular, prior practice of the items in the lexical decision task might have strengthened the weaker language to a greater extent than the stronger language, leading to a change in their dominance relation in the picture naming task. However, a comparison with a similar study suggests that the results obtained in the picture naming task are likely to be due to the nature of the task rather than to different practice effects for the two languages. Specifically, in a recent study by Mosca and Clahsen (2016), language switching costs were measured in a group of unbalanced bilinguals (L1 German–L2 English) performing a bilingual picture naming task. Participants were classified as highly proficient L2 speakers (C1 level of the CEFR), scoring 75.4% in the Oxford Placement Test. Despite the proficiency difference, results revealed symmetrical switching costs for the two languages and a tendency for paradoxical language dominance. In this picture naming task, there was no prior practice of the items. Based on this as well as previous evidence (e.g., Christoffels et al., 2007) it seems that in language production, symmetrical switching costs and paradoxical language dominance are to be expected in unbalanced bilinguals–highly proficient L2 speakers. Importantly, compared to Mosca and Clahsen (2016), the bilinguals tested in the present study were more proficient L2 speakers, scoring 84.4% at the Oxford Placement level (C1-C2 level of CEFR). This suggests that the pattern of results, namely symmetrical switching costs and paradoxical language dominance, found in the present study can be confidently attributed to the task itself together with the high proficiency reached in the L2 and less so by the fact that the weaker language might have benefited more than the stronger language from prior practice. Regardless, further studies are needed to assess the effect of practice on language control (see also Branzi et al., 2014; Declerck and Philipp, 2017).

Thus, why did we find ceiling performance in the production, but not in the recognition task? The reason might lie in the difference between the processes supporting language production and recognition. Indeed, while language recognition is mostly supported by a bottom-up mechanism, language control in production is a mainly top-down process (see next session for further discussion on bottom-up vs. top-down control). As Yeung and Monsell (2003) observed, top-down control is effortful and, therefore, minimized where possible. In particular, Monsell et al. (2000) suggested that greater inhibition on the stronger task might be a useful strategy only when the activation strength of two tasks is extremely unequal, but not when the difference does not reach a certain threshold. Therefore, it might be the case that symmetrical switching costs between a stronger and a weaker language in language production are due to a process that aims at minimizing the effort.

Furthermore, Monsell et al. (2000) showed that more activation for the stronger than for the weaker task refers to languages' activation level at the beginning of the experiment, but that this state can be changed by practicing the tasks during the experiment. Based on this, an alternative interpretation of the data might be that the symmetrical switching costs could be attributed to a change in the amount of language suppression over time. Specifically, while at the beginning of the task the stronger language is suppressed more because it is perceived as more activated than the weaker, after a certain amount of practice, the actual strength of language activation might be perceived (the weaker language is activated more than otherwise), leading to larger switching costs for the weaker than for the stronger language. Such a modulation of language inhibition over time would yield overall symmetrical switching costs. To address this issue, however, future studies need to investigate whether the way languages are controlled remains unvaried for the duration of the task or whether the equilibrium between the languages is affected by the practice.

#### Single-language block

In the single-language block, we found that responses in the L1 were on average faster than in the L2, but that the difference was not significant. Comparable performance in the two languages might indicate that our participants were fairly balanced bilinguals. However, this assumption is in contrast to their performance in the lexical decision task, where participants responded faster to L1 words than L2 ones.

Similar performances in L1 and L2 might be due to the order in which languages were presented in the single-language block. Recall that half of the participants named pictures first in the L1 and then in the L2 and the other half named pictures in the opposite order. Several studies investigating the effect of language order in language production have shown that, presented two separate language blocks, L1 naming is hampered if preceded by L2 naming. However, this negative effect is not found in the L2, where performance seems to benefit from the previous naming in the L1 (Levy et al., 2007; Misra et al., 2012; Branzi et al., 2014). One way to interpret this asymmetry is by assuming that during L2 naming, the L1 is strongly inhibited and that the inhibitory carry-over effect hampers L1 naming in the subsequent block. During L1 naming, however, weak or no inhibition is applied to the L2, leading to positive priming when the same pictures have to be named in a following L2 block (Misra et al., 2012). Therefore, negative priming on the L1 and positive priming on the L2 might explain, why we do not detect a reliable dominance effect in our group of bilinguals in the single-language block.

#### Mixing costs

Concerning language mixing costs, we found that trials of the single-language block were responded faster than repetition trials of the mixed-language block. The difference, however, was present only in the L1, but not in the L2, i.e., we found asymmetrical mixing costs. This effect is to be attributed to the fact that while in the single-language block, L1 and L2 were responded equally fast, in the mixed-language block, L1 responses became slower than L2 ones. The paradoxical dominance effect was mirrored by the accuracy data, showing similar error rates for the L1 and the L2 in the single-language block, and more errors for the L1 than for the L2 in the mixed-language block.

Better performance in the weaker than in the stronger language in the mixing language context is not a novel finding in language switching research (e.g., Costa and Santesteban, 2004; Costa et al., 2006; Christoffels et al., 2007; Schwieter and Sunderman, 2008; Verhoef et al., 2009, 2010). The paradoxical advantage of the weaker over the stronger language has been interpreted as speakers' unconscious strategy to help the weaker language, by lowering the activation level of the stronger language (e.g., Meuter, 2005; Christoffels et al., 2007). This effect can be attributed to proactive control that aims at anticipating and preventing interference before they occur (Braver, 2012). We suppose that, in the present study, interference from the stronger L1 was prevented by lowering its overall activation level (slower and less accurate performance in L1 than in L2). Differently from proactive control, reactive control is recruited as a late correction mechanism that activates only after a change has occurred (Jacoby et al., 1999), such as a language switch. In this view, after a language change is detected, the non-relevant language is reactively inhibited, leading to switching costs when that language needs to be reactivated (Green, 1998).

### Picture naming vs. lexical decision task

In the production task, we found that L2 responses were named faster than L1 ones. This pattern has been interpreted as speakers' unconscious strategy to help the weaker language, by making the stronger language less available. This is not what we found in the recognition task, where words were responded to faster in the L1 compared to the L2. One might suppose that the different dominance effect found in reception and production might depend on task goals. While in the production task, the goal of the task was to name pictures in one or the other language, in the reception task, the aim of the task was to decide whether a string of letters was a real word or not irrespective of language membership. Therefore, it may be the case that bilingual language control relies on strategies to regulate languages' activation strength only when language membership is relevant for the task. However, previous bilingual lexical decision studies using a language-specific paradigm (i.e., deciding whether a string of letters is a word in a determined language) do not report faster responses in the weaker than in the stronger language (e.g., von Studnitz and Green, 1997; Thomas and Allport, 2000). This suggests that language membership is not the reason why paradoxical language dominance occurs. More generally, we are not aware of any recognition study with bilinguals reporting better performance in the weaker than in the stronger language. This indicates that the paradoxical language effect seems to be limited to bilingual production, and not to bilingual recognition.

Language production and language recognition are, indeed, two different processes. While in language production, information mainly flows in a top-down fashion (from the concept to the lemma, down to the phonological and then to the articulatory level), language recognition is a predominantly bottom-up process (from letters, up to word recognition, to the phonological representation and then to the concept level). This difference was already captured in a paper describing the developmental Bilingual Interactive Activation (BIA-d) model by Grainger et al. (2010). According to an interpretation of the BIA model provided by these authors, the relevant language node is activated top-down in production and bottom-up in recognition. With concern to production, the authors postulated that words belonging to the stronger language (e.g., L1) require more top-down inhibition when a word in a weaker language (e.g., L2) is produced than vice versa. Similar to what suggested by the IC model, also this interpretation of the BIA model predicts larger switching costs for the stronger than for the weaker language. As to language recognition, Grainger and colleagues assumed that, due to their higher resting level of activation, when L1 words are presented, words of the stronger L1 will send more bottom-up input to the L1 language node compared to L2 words to the L2 language node, when L2 words are presented. Because of this difference, in the case of language switching, interference from the stronger language will be greater to overcome than vice versa, leading to larger switching costs for the weaker compared to the stronger language. Unfortunately, the results obtained in the present study do not support this hypothesis, in that language switching costs were symmetrical in the production task and larger for the stronger than the weaker language in the recognition task.

A way to understand the processes underpinning language control in production and recognition is by considering that top-down and bottom-up attentions are commonly deemed as two different types of information processing (e.g., Carrasco, 2011; Pinto et al., 2013). Top-down attention is goal-oriented, meaning that attention is voluntary allocated to certain features (e.g., Beauchamp et al., 1997). Bottom–up attention is stimulus-driven, indicating that certain stimuli can attract attention, even though the subject is not doing so intentionally (e.g., Schreij et al., 2008). Thus, the main difference between top-down and bottom-up attention is that the former is voluntary/non-automatic, while the latter is involuntary/automatic. Moreover, top-down attention is supposed to be more flexible than bottom-up attention (e.g., Shiffrin and Schneider, 1977). Specifically, the hypothesis is that since top-down attention is voluntary, resources can be strategically allocated depending on the task (e.g., Kinchla, 1980; Jonides, 1981; Giordano et al., 2009).

Because of this, one might suggest that while in production, bilingual language control predominantly relies on a flexible and strategic top-down mechanism, in recognition, bilingual language control is mainly supported by a more rigid bottom-up process. The flexible nature of the top-down control could explain why we found paradoxical dominance effect (interpreted as a strategy to prevent interference from the stronger language) in the production, but not in the recognition task.

Similarly, the different language switching pattern found in the recognition and the production task could be ascribed to the difference between top-down and bottom-up mechanisms. In the recognition task, asymmetrical switching costs might be attributed to the fact that since words are more activated in L1 than in L2, L1 words need to be inhibited more than L2 words, leading to larger reactivation costs for the L1 relative to the L2. In the picture naming task, symmetrical switching costs for the stronger and the weaker language could be explained either by a strategic process of costs' minimization (i.e., similar amount of inhibition on L1 and L2) or by a modulation of language inhibition over time (stronger inhibition on L1 than L2 at the beginning of the task, but reversed inhibition pattern afterwards). However, more studies are needed to assess whether the difference between language control in production and recognition are to be attributed to the fact that they are mainly supported by two different mechanisms of information processing. A more systematic way to address this issue would be, for example, by including a single-language block for the recognition task, so to compare language mixing costs in recognition and production. Alternatively, it could be useful to use a language-specific recognition task, in which only words are used and where speakers have to decide whether a given word belongs to either L1 or L2. This type of task would be more comparable to a picture naming task since only existing words are used and language membership is relevant for the task.

## Conclusion

The aim of this study was to investigate bilingual language control in reception and production. In particular, we asked whether language control in bilingual perception relies on the same mechanism as in bilingual production and whether these two processes can be incorporated into a single bilingual language control model. To investigate this issue, we considered two prominent models of bilinguals language control, according to which, when one language is in use, the other is suppressed. The (IC) model (Green, 1986, 1993, 1998) suggests that the amount of inhibition applied to the non-relevant language depends on its dominance (the stronger the language the greater the inhibition). The Bilingual Interactive Activation (BIA) model (Grainger and Dijkstra, 1992; Dijkstra and van Heuven, 1998; van Heuven et al., 1998), proposes that inhibition from the stronger to the weaker language is greater than the other way around (the stronger the language the weaker the inhibition). Therefore, in the case of language dominance difference, both types of models predict a different amount of inhibition for the two languages and, consequently, a different amount of reactivation costs (asymmetrical switching costs). For the IC model switching costs are dominance-related (the stronger the language, the greater the cost), whereas for the BIA model switching costs are dominance-reversed (the greater the language, the smaller the cost). Based on previous findings, we also considered the possibility that when the difference between L1 and L2 dominance level is relatively small, the magnitude of inhibition applied to the two languages might be comparable (symmetrical switching costs).

To investigate the mechanisms underpinning language control in production and recognition, we tested native speakers of Dutch (L1)-highly proficient speakers of English (L2) in a lexical decision and a picture naming task with language switching. Participants reported being more proficient and more exposed to the L1 compared to the L2. Results from the bilingual lexical decision task confirmed that L1 and L2 were processed differently. Specifically, we found that language switching was costly only for the L1 but not for the L2. We suggested that when L2 words are presented, L1 competitors need to inhibited. If during L2 processing, L1 words are suppressed, reactivating the L1 on a switch trial will lead to L1 switching costs. However, when L1 words are presented, the target word is fast recognized and words from the weaker L2 will not have enough time/strength to compete. In this case, the inhibition applied to the weaker L2 competitors will be relatively small or absent, yielding to undetectable L2 switching costs when the L2 becomes relevant again.

These results are in contrast with the BIA model, according to which switching costs are dominance-reversed, but they are in line with the dominance-related IC model, in that we measured switching costs in the stronger L1, and only a tendency toward switching cost in the L2.

In contrast to the lexical decision task, in the picture naming task we measured the same amount of switching costs for the L1 and the L2. Symmetrical switching costs between a stronger and a weaker language cannot be accounted for by a conservative dominance-related account of language control. We suggested that symmetrical switching costs in bilinguals with a relatively small difference between L1 and L2 proficiency level might be related to speakers' temporary adjustment to the task. Specifically, we assume that in the production task, the language control system strategically adapts to the task either by minimizing the effort of switching between languages (i.e., applying a comparable amount of inhibition on L1 and L2) or by adjusting the amount of language inhibition during the task (greater inhibition on L1 than L2 at the beginning of the task, and greater inhibition on L2 than L1 toward the end of the task). Importantly, whilst it remains unclear whether language switching costs can be considered a direct test toward or against a specific model (e.g., Bobb and Wodniecka, 2013; Baus et al., 2015), they might still be informative about the general nature of the processes. As a sizable number of studies have shown, language switching costs very much depend on the type of task. Because of this, it might become difficult to provide unambiguous evidence toward or against a specific model. However, the fact that two different tasks give rise to different performances, implies that language control is not a fixed mechanism and can be influenced by the context. In the present paper, participants were asked to perform a language switching task in two different contexts, i.e., in a recognition and a production setting. Results showed not only different language switching pattern between the two tasks but also a paradoxical language effect (faster responses in the L2 than in the L1) in the production but not in the recognition task. Although it remains unclear to what extent these results are informative with regard to specific models of language control, they clearly reveal that the two tasks were performed differently. We think that this result represents an important starting point to bring awareness about the fact that language control is a much more flexible mechanism than previously believed and that because of its malleable nature it is difficult to circumscribe it within a specific model.

## Ethics statement

This study was carried out in accordance with the recommendations of the Ethics Committee of the University of Potsdam with written informed consent from all subjects. All subjects gave written informed consent in accordance with the Declaration of Helsinki. The protocol was approved by the Ethics Committee of the University of Potsdam.

## Author contributions

MM: Conception, design, and programming of the experiment. Acquisition, analysis, and interpretation of the data. Draft and revision of the manuscript. Final approval of the version to be published and of the content and the accuracy or integrity of any part of the work. KdB: Conception of the experiment. Draft and revision of the manuscript. Final approval of the version to be published as well as of the content and the accuracy or integrity of any part of the work.

### Conflict of interest statement

The authors declare that the research was conducted in the absence of any commercial or financial relationships that could be construed as a potential conflict of interest.

## Footnotes

^1^In the extended version of the BIA model, the BIA+ model (Dijkstra and van Heuven, 2002), the asymmetrical inhibition from language node to words is removed. As replacement to the language nodes, the BIA+ model introduces a task/decision system that should perform the same functions. However, how the mechanism exactly works is not specified, leaving the BIA+ model an incomplete model of bilingual processing (see also Bultena and Dijkstra, 2012).

^2^In the lexical decision task, we used words representing the same objects in L1 (Dutch) and L2 (English) in order to be consistent with the picture naming, where the same pictures were used to test performance in the two languages
