# Supplementary material for: Bilingual Language Switching: Production vs. Recognition
Source: Front Psychol. 2017 Jun 7;8:934. doi: 10.3389/fpsyg.2017.00934 (PMC5461355; doi:10.3389/fpsyg.2017.00934)
Supplement: Supplementary file 1 [file DataSheet1.docx]

**Bilingual Language Switching: Production vs. Recognition**

Michela Mosca^1*^, Kees de Bot^2^

^1^International Doctorate in Experimental Approaches to Language And Brain (IDEALAB), University of Potsdam, Germany; University of Groningen, The Netherlands; University of Trento, Italy; Newcastle University, United Kingdom and Macquarie University, Australia

^2^ University of Pannonia, Hungary

*** Correspondence:**Michela Mosca
[mosca@uni-potsdam.de](mailto:mosca@uni-potsdam.de)

# Supplementary Data

## **Appendix A**. Participants

All participants were native speakers of Dutch (L1), late learners of English (L2). Twenty-five of them reported knowing an additional language, nineteen had knowledge of two additional languages and fifteen knew three additional languages. German was one of those languages for seventeen participants, French for fifteen, Spanish for eleven, Swedish for five, Frisian for three, Italian for two, Greek, Latin and Low Saxon for one person respectively. Six of them reported being fluent in speaking an additional language and four of them in reading in an additional language. All participants considered themselves fluent speakers and readers of both L1 (Dutch) and L2 (English). Below are reported the information about the self-assessed language history and competence in L1 (Dutch) and L2 (English) from the Language Experience and Proficiency Questionnaire (LEAP-Q).

Table A1. Mean scores (standard deviation in brackets) about language age of acquisition (AoA) and age (in years) when participants became fluent in L1 (Dutch) and L2 (English).

|  | **AoA** | **Speaking fluency** | **Reading AoA** | **Reading fluency** |
| --- | --- | --- | --- | --- |
| **L1** | 0 | 5.74  (2.15) | 5.11  (1.28) | 8.66  (2.01) |
| **L2** | 9.35  (2.34) | 16.74  (2.37) | 11.16  (2.36) | 16.60  (2.04) |

Table A2. Mean scores (standard deviation in brackets) of the self-rating task based on a ten-point scale (0= no knowledge, 10= perfect knowledge) for speaking, comprehension and reading skills in L1 (Dutch) and L2 (English).

|  | **Speaking** | **Comprehension** | **Reading** | **Mean** |
| --- | --- | --- | --- | --- |
| **L1** | 8.82  (0.98) | 9.50  (0.69) | 9.17  (0.90) | 9.16  (0.85) |
| **L2** | 6.96  (0.96) | 8.06  (1.14) | 7.93  (1.04) | 7.65  (1.04) |

Table A3. Mean scores (standard deviation in brackets) relative to the amount of current language exposure in L1 (Dutch) and L2 (English) based on a ten-point scale (0= never exposed, 10= always exposed) in different contexts (interaction with friends and family, during reading, watching TV, listening to the radio, self-instruction and language tapes).

|  | **Friends** | **Family** | **Watching TV** | **Listening to radio/music** | **Reading** | **Language lab/ Self-instruction** |
| --- | --- | --- | --- | --- | --- | --- |
| **L1** | 7.75  (1.71) | 8.46  (2.45) | 5.10  (1.98) | 3.60  (2.24) | 4.57  (2.48) | 1.50  (2.44) |
| **L2** | 4.65  (2.67) | 0.79  (1.39) | 7.42  (1.64) | 6.75  (2.50) | 7.41  (1.84) | 2.89  (3.05) |

Table A4. Mean scores (standard deviation in brackets) relative to the amount (in years) of prior language exposure in L1 (Dutch) and L2 (English) in different environments (in a country, in a family, at school/work).

|  | **Country** | **Family** | **School/Work** |
| --- | --- | --- | --- |
| **L1** | 21.39  (4.57) | 20.24  (1.49) | 18.66  (5.44) |
| **L2** | 0.38  (1.54) | 0  (0) | 2.55  (3.73) |

Table A5. Mean scores (standard deviation in brackets) relative to the amount of contribution of different factors (interaction with friends and family, reading, watching TV, listening to the radio, self-instruction and language tapes) in learning L1 (Dutch) and L2 (English). Scores are based on a ten-point scale (0= not a contributor, 10= most important contributor).

|  | **Friends** | **Family** | **Watching TV** | **Listening to radio/music** | **Reading** | **Language lab/ Self-instruction** |
| --- | --- | --- | --- | --- | --- | --- |
| **L1** | 7.96  (1.23) | 8.37  (1.89) | 6.10  (1.51) | 3.89  (2.56) | 7.58  (1.23) | 1.82  (2.46) |
| **L2** | 5.20  (3.02) | 1.46  (2.45) | 7.93  (1.33) | 4.89  (2.50) | 7.96  (3) | 3.63  (3.10) |

# Supplementary Data

## Appendix B. Materials (L1 Dutch, L2 English)

List of the Words/Picture names (a) and Pseudowords (b) used:

a) *Bril, glasses; Borstel, brush; Citroen, lemon; Fles, bottle; Handschoen, glove; Horloge, watch; Jurk, dress; Kers, candle; Knop, button; Lepel, spoon; Potlood, pencil; Schommel, swing; Vogel, bird; Wolk, cloud*.

b) *Bris, smartes; Garstel, brunk; Cichoon, tewon; Snes, boddle; Handspleen, wrove; Melloge, wamps; Jark, pless; Hers, bantle; Blop, cunton; Remel, spean; Petloog, runcil; Scharmel, pring; Bosel, bime; Wolm, croud.*

# Supplementary Data

## **Appendix C.** Data Analysis

Visual inspection revealed that the removal of the extreme values did not result in normality, violating the normality assumption underlying the general linear model (Baayen & Milin, 2010). Tests for skewness indicated that the data were positively skewed (i.e. data skewness > 0.80). To decide on a suitable transformation, we estimated the lambda parameter of the Box-Cox transformation, which ensures that residuals RTs are approximately normal (Box & Cox, 1964). Lambda values of -0.50, -0.14 and -0.30 (for lexical decision, and single- and mixed-language picture naming) indicated that a reciprocal square root and a log transformation was appropriate for the lexical decision and the picture naming task, respectively. Visual inspections of the data together with the skewness tests confirmed the reciprocal square root and the log transformation as appropriate to approximate normality in the two tasks (data skewness < 0.15).

All the analyses were carried out in GNU-R version 3.2.2 (R Core Team, 2015) using the lme4 package version 1.1-9 (Bates et al., 2015). Reaction times data were fitted in linear mixed effects models, while accuracy binary data (1= correct, 0= incorrect) were fitted in generalized linear mixed effects models with a logistic link function.

All models included crossed random effects for participants and items. The best-fit models were selected using a forward stepwise procedure, where only significant predictors were used to compare nested models of increasing complexity. During models comparisons, parameters were estimated using the Maximum Likelihood for fixed effects and Restricted Maximum Likelihood for random effects comparisons (Pinheiro & Bates, 2000). Models that failed to converge were excluded from comparisons of further models. The quality of the fit was determined by the Akaike's information criterion (AIC; Akaike, 1998) and its significance by the likelihood ratio test (see also Matuschek et al., 2015).

Based on this criteria, the best-fit model for the lexical decision data included the experimental factors of Language (L1 vs. L2), Condition (Repetition vs. Switch), Word Type (Word vs. Pseudoword) and Word Type Change (Yes vs. No) as significant predictors. The model had fully random slopes and intercepts by participants and items structure (the so-called maximal model, [Barr et al., 2013](http://journal.frontiersin.org/article/10.3389/fpsyg.2016.00403/full#B1); [Bates et al., 2015](http://journal.frontiersin.org/article/10.3389/fpsyg.2016.00403/full#B3)). Main effects and interactions were coded using sum contrasts (i.e. -0.5 vs. +0.5). For the picture naming task, the best-fit model included the experimental factor of Language (L1 vs. L2) and Block (Single vs. Mixed). In addition to this, the factor Condition (Repetition vs. Switch) was included in the mixed-language block. The models had random intercepts by participants (for the factor Language) in the single-language block and by participants and items (for the factor Condition) in the mixed-language block. Before accepting the models, we checked whether the they provided a satisfactory fit to the data, i.e. “models’ criticism”. Visual inspection revealed that the distribution of the residuals tended to have a thicker right tail than expected for a normal distribution and that variance was not uniformly distributed (see also Baayen, 2008). To approximate residuals normality and to stabilize variance (“homoscedasticity”), we removed standardized residuals larger than 2.5 (see also Baayen, 2008). Mild a-priori screening for outliers together with model criticism are considered two essential procedures within mixed-modelling approach to avoid model distortion (Baayen & Milin, 2010).
